# Molecular characterization and construction of an infectious clone of a pepper isolate of *Beet curly top Iran virus*

**Published:** 2016-06

**Authors:** Omid Eini, Ghazal Ebadzad Sahraei, Seyed Ali Akbar Behjatnia

**Affiliations:** 1 Department of Plant Protection, School of Agriculture, University of Zanjan, Zanjan, Iran; 2 Plant Virology Research Center, College of Agriculture, Shiraz University, Shiraz, Iran

**Keywords:** Agroinoculation, BCTIV, Geminivirus, Pepper

## Abstract

Geminiviruses cause curly top disease, in dicotyledonous plants which constrains host crop production. *Beet curly top Iran virus *(BCTIV) is a widespread *Becurtovirus *(family *Geminiviridae*) in numerous areas within Iran. In this study, we isolated and analyzed a full-length genomic DNA of a new variant of BCTIV from pepper crops in the Kaftark region, east of Shiraz (proposed acronym: BCTIV-Kaf [IR: Kaf:2016:Pepper]). Infected pepper plants showed shortening of internodes, severe interveinal chlorosis, upward leaf rolling and leaf curling. Sequence and phylogenetic analysis showed this BCTIV variant grouped with sugar beet isolates of BCTIV and has the highest similarity to a sugar beet BCTIV isolate from Negar town in Kerman province, Iran. It was more distantly related to a bean isolate of BCTIV from northeast region of Iran. A tandem repeat partial dimmer of BCTIV was constructed and found to be infectious in pepper, tomato and *Nicotiana benthamiana *plants. Results of this study indicated that BCTIV-Kaf is a new variant of BCTIV infecting pepper plants in Shiraz and that geographic location rather than the type of host plant has more effect on genetic diversity of BCTIV in Iran.

## INTRODUCTION

Geminiviruses have a major impact on crop production worldwide. They are characterized by their twinned icosahedra particles and their single-stranded DNA genome. Recently family *Geminiviridae *was grouped into seven genera including *Curtovirus *and *Becurtovirus *[1]. *Beet curly top Iran virus *(BCTIV) and *Spinach curly top Arizona virus *(SCTAV) are members of *Becurtovirus *[1].

BCTIV and a severe strain of *Beet curly top virus *(BCTV-Svr) from *Curtovirus *genus [2], have been reported to induce curly top disease in dicotyledonous plants in various regions of Iran [3, 4]. BCTIV is a major pathogen in the sugar beet and has been recently reported in other crops such as beans (*Phaseolus vulgaris*) and cowpeas (*Vigna unguiculata*) [5].

The genome organization of BCTIV is different from *Beet curly top virus *(BCTV), the type member of *Curtovirus *genus. The full-length sequence of BCTIV also has low level of identity to the genome of curtoviruses. BCTIV has only five open reading frames (ORFs), three on the virion-sense strand (called V1, V2 and V3) which are closely related to the corresponding ORFs of curtoviruses; while the other two ORFs on the complementary-sense strand (called C1 and C2) are quite different and only distantly related to those of mastreviruses [4]. This compares with the genome of BCTV and other curtoviruses, which contain seven ORFs, including V1, V2 and V3, on the virion-sense strand and C1, C2, C3 and C4 on the complementary-sense strand [2]. The genome of curtoviruses has a large intergenic region (IR) which includes a sequence capable of forming a stem-loop structure containing a universally conserved nonanucleotide motif (TAATATT/AC) [6] while the BCTIV genome comprises two IRs. The BCTIV large IR has a novel nonanucleotide (TAAGATT/CC) within the stem- loop structure with a unique nick site [4].

Preparing an infectious clone from the viruses would lead to better understanding of virus-plant interaction. This clone would be used as a tool for reverse genetic research and also to screen host plants for resistance phenotypes in breeding programs. The infectious clone can be delivered into host plants/cells using an *Agrobacterium *system and particle bombardment [7].

Construction of two infectious clones of BCTIV, both originated from infected sugar beets, has already been reported [8, 9]. No other infectious clone from other infected sources of BCTIV has been constructed yet and no accession number for full- length genome sequences of infected pepper plants has been deposited into GenBank. The aim of this study was to characterize a pepper isolate of BCTIV, to construct the infectious clone of this isolate and to test the infectivity of the cloned genome of this isolate on three different plant species.

## MATERIALS AND METHODS

**Plant material: **Leaf samples were collected from pepper plants showing yellowing and leaf curling symptoms in pepper fields in the Kaftarak region, east of Shiraz, Fars province, Iran. Pepper (*Capsicum frutescens*), tomato (*Solanum lycopercicum*) and *Nicotiana benthamiana *plants were grown in pots containing loamy sand, vermiculite, and coco peat (1:1:1). Plants were maintained in an insect-free greenhouse under 14/10- h light/dark periods, 24±3 °C and 85 % relative humidity.

**DNA extraction, cloning and sequencing of the viral genome: **Total DNAs were extracted from leaf tissues by a modified CTAB method [10]. DNAs were tested for BCTIV infection by polymerase chain reaction (PCR) using a BCTIV specific primer pair BCTIV-F/BCTIV-R (Table 1) designed to specifically amplify a portion of the coat protein gene [11]. A DNA fragment approximately 700 bp in size was amplified. This fragment was cloned into a pGEM-T vector and sequenced (Macrogen, Korea). To amplify the full-length DNA genome of the virus, the DNA of a naturally infected pepper plant from Kaftarak was used in PCR along with a specific adjacent primer pair (BCTIV-F1/ BCTIV-R1, Table 1), flanking the naturally occurring *Hind *III restriction sites, which were designed from the sequence information from the amplified coat protein fragment. PCR products were subjected to electrophoresis in 1.2 % agarose gel. The full-length PCR fragments from an infected plant were excised from the agarose gel and purified using QIAquick Gel Extraction Kit (Qiagen) according to the manufacturer's protocol. The purified fragments were cloned into the PGEM -T vector (Promega). Three individual clones were sequenced using the universal vector primers, T7 and SP6. The obtained sequences were used to design new primers including BCT1 F/R and BC2 F/R primer pairs (Table 1) to sequence the other parts of the cloned full length DNAs in PGEM -T using the primer walking technique.

**Table 1:** Oligonucleotide primers used in this study

Primers	Size (nt)	Sequences (5′ to 3′)*
BCTIV-F	17	TACAAGTATGGCGGTTC
BCTIV-R	21	GAGTAAAGCATTCTCCTTCAC
BCTIV- F1	25	CCAAGCTTAAGGTTAGTTTTAAGCG
BCTIV-R1	26	AAAAGCTTCAGCAATTTCTTCACTTC
BCT1- F	17	CCGACTCAGTTGAGTAC
BCT1- R	19	CAGGAGCATGTTTGTTGTG
BC2- F	21	CCGACTCAGTTGAGTACTATC
BC2- R	21	GACTCTGAACCGCCATACTTC

* *Hind *III restriction site underlined

**Sequence analysis: **The obtained sequences were compared to the available sequences in the GenBank database (www.ncbi.nlm.nih.gov/gen bank) using BLAST software. A contig of the sequences obtained for the full-length fragments was prepared (BioEdit, V7.2.5, Carlsbad CA). The sequences from three individual clones containing the full-length PCR fragments were aligned to obtain the consensus sequence. Among them, one full-length monomer genome clone that shares the highest sequence homology with the consensus sequence designated pGem-1.0BCTIV-Kaf and used as a template to construct the infectious clone of BCTIV-Kaf (see below).

For phylogenetic analysis, multiple sequence alignment for sixteen BCTIV isolates from various hosts and regions was achieved using the program AlignX (BioEdi) with the ClustalW algorithm [12]. Then a neighbor-joining method was used to construct the phylogenetic tree with 1000 bootstrap replications using MEGA 6 [13].

To investigate the variability of proteins encoded by BCTIV-Kaf, we aligned the amino acid sequences for each ORF and compared them to the ORFs of BCTIV isolates reported from various hosts and regions.

**Construction of an infectious clone of BCTIV-Kaf: **To construct a head-to-tail partial dimmer of BCTIV, a 1028 bp *Hind*III/*EcoR*I fragment was released from the pGem-1.0BCTIV-Kaf and constructed through digestion of this clone with *Hind*III/*EcoR*I enzymes. This fragment was sub-cloned into corresponding sites of a binary vector, pBin20 [14], to obtain the pBin20-0.4BCTIV-Kaf construct. The full- length *Hind*III monomeric DNA was released from the pGem-1.0BCTIV-Kaf construct through digestion of this clone with *Hind*III and then sub-cloned into the corresponding site of pBin20-0.4BCTIV-Kaf construct to create a 1.4 mer DNA construct of BCTIV that designated pBin20-1.4BCTIV-Kaf. The resulting construct was introduced into *Agrobacterium tumefaciens *strain C58 by electroporation with a Gene Pulser apparatus (Bio-Rad, Germany) according to the manufacturer's specifications.

**Virus infectivity and hybridization assay:** *A. tumefaciens *cells harboring pBin20- 1.4BCTIV-Kaf were grown at 28ºC for 48 h and then inoculated into the axillary buds of pepper (bird’s eye chili, *C. frutescens*, a local variety), tomato (*S. lycopersicum *cv. Grosse lisse) and *N. benthamiana *seedlings at the four-leaf stage, as described previously [15]. The agroinoculated plants were maintained in a greenhouse at 24 ±3 ºC (14:10 h, light:dark) and evaluated for symptom appearance at 14 days post-inoculation (dpi). Developing leaves were sampled from these plants 21 dpi and DNA was extracted and analyzed for the presence of the viral genome by Southern blot hybridization using a BCTIV-Kaf specific DNA probe obtained by releasing a DNA fragment (1028 pb in length) from pGem-1.0BCTIV-Kaf following digestion of this plasmid with *Hind*III/*EcoR*I. The released DNA fragment was labeled by incorporation of 32P- labeled dCTP using a DNA labeling kit (Roche labeling kit) and then used for hybridization [16].

## RESULTS

**Figure 1:**
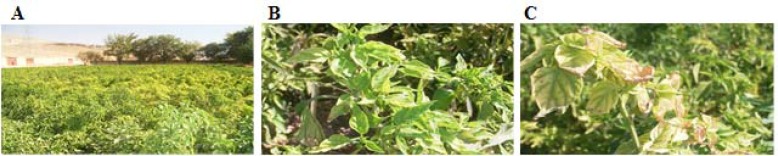
Symptoms of yellowing and curly top disease in pepper crops in Kaftarak region, Shiraz, Fars province, Iran. (A) An infected pepper field. Yellowing is the dominant symptom. (B) Stunting, shortening of the leaf nodes, leaf curling and upward leaf rolling of pepper plants. (C) Severe interveinal yellowing with marginal leaf necrosis in severe infected pepper crops

In pepper fields in Kaftarak region of Shiraz that is the major area of pepper cultivation in Fars province, yellowing was the dominant symptom (Fig. 1A). Other symptoms of the infected plants included stunting, shortening of the leaf nodes, leaf curling and upward leaf rolling (Fig. 1B). Severely infected plants showed severe interveinal chlorosis with marginal leaf necrosis (Fig. 1C).

**Figure 2:**
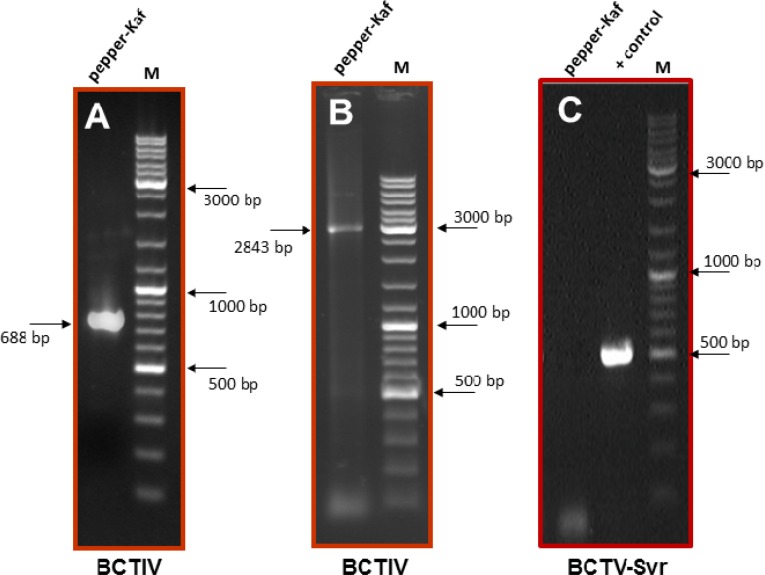
Electrophoretic pattern of the 682 (A) and 2843 (B) bp PCR product of BCTIV-Kaf amplified from a naturally infected pepper plant from Kaftarak region, East of Shiraz, using two specific primer pairs BCTIV-F/R (A) and BCTIV-F1/R1 (B), respectively. No amplified DNA was obtained from the same plant (C, lane 1) when a specific primer pair of BCTV-Svr (358V/853C, [17]) was used in PCR, while an approximately 500 bp fragment (C, lane 2) was amplified from a BCTV-Svr cloned DNA [17] used as a positive (+) control. M=marker

DNA extracts obtained from these plants were analyzed by PCR for the presence of BCTIV.By using the specific primer pair BCTIV-F/BCTIV-R (Table 1), a DNA fragment of the expected size (approximately 700 bp) was amplified from all symptomatic plants (Fig. 2A). However, by using the specific primer pair for the Iranian isolate of BCTV-Svr [3] in PCR, no amplified DNA was obtained from symptomatic pepper plants collected in the Kaftark region in this study (Fig. 2C), indicating that these plants were infected by BCTIV.

The size of the BCTIV fragment amplified from naturally symptomatic pepper plants was determined to be exactly 688 bp when cloned and sequenced. The sequence of this DNA fragment was used to design a specific adjacent primer pair, BCTIV-F1/R1 (Table 1), which gave a single PCR product of the size predicted for the full-length genome of the virus in PCR (Fig. 2B). The sequences of both strands of three independent clones of full-length genome were obtained. A contig of these sequences was prepared and the consensus sequence of the BCTIV full-length genome was obtained to be 2843 bp. The nucleotide sequence data have been deposited in GenBank under accession number KP410285.

The features of the BCTIV-Kaf genome are similar to those of the other BCTIV isolates [4, 9] which contains three ORFs on the virion-sense strand and two ORFs on the complementary-sense strand. A large (296 nt) and a small (245 nt) IR were also present in the genome of BCTIV-Kaf. The large IR of the virus embodies a stem-loop structure and the loop contains the TAAGATT/CC sequence with the T/CC cleavage site where rolling circle replication is initiated [25]. .

Comparison of complete DNA nucleotide sequence of the BCTIV-Kaf genome with those of other BCTIV isolates which complete genome information were available (Table 2) indicated that this BCTIV isolate shared 88.0–99.3 % sequence identity (Table with the previously described BCTIV isolates [4, 5, 9]. BCTIV-Kaf is more similar to a sugar beet isolate of BCTIV (Acc. No. JQ707948) from Negar town in Kerman Province [8] with their genome sharing 99.3 % homology. It was more distantly related to a bean isolate of BCTIV from Neyshabour, Khorasan Razavi province, northeast region of Iran [5] as their genomes share only 88.0 % homology (Table 3). According to ICTV criteria, BCTIV-Kaf can be classified as a variant of BCTIV.

**Table 2:** Complete the name of BCTIV isolate of this study as mentioned in ABSTRACT. i. e., [IR:Kaf:2016:pepper

Isolate name	GenBan	Host plant	Location/Province*	Reference
BCTIV- BCTIV [IR:Boj:3B:Sug:10]	JX94557	*Capsicum frutescens Beta vulgaris*	Fars (S) Khorasan (NE)	This study [5]
BCTIV[IR:Yaz:B15P:Sug:06]	JQ70793	*Beta vulgaris*	Yazd (C)	[8]
BCTIV [IR:Shi:B18 K:Sug:06]	JQ70793	*Beta vulgaris*	Fars (S)	[8]
BCTIV [IR:Kav:B22 K:Sug:08]	JQ70794	*Beta vulgaris*	Fars (S)	[8]
BCTIV[IR:Neg:B25P:Sug:08]	JQ70794	*Beta vulgaris*	Kerman (SE)	[8]
BCTIV [IR:Yaz:B35 K:Sug:06]	JQ70795	*Beta vulgaris*	Yazd (C)	[8]
BCTIV [IR:Siv:Sug:2]	JX08225	*Beta vulgaris*	Fars (S)	[9]
BCTIV-A [IR:Neg:B32P:Sug:08]	JQ70794	*Beta vulgaris*	Kerman (SE)	[8]
BCTIV-A [IR:Hom:Tu69:sug:10]	JX98767	*Beta vulgaris subsp.*	Fars (S)	[5]
BCTIV-B	JX96623	*Solanum lycopersicum*	Khorasan Razavi (NE)	[5]
BCTIV-B	JX13163	*Vigna unguiculata*	Khorasan Razavi (NE)	[5]
BCTIV-B	JX13163	*Vigna unguiculata*	Khorasan Razavi (NE)	[5]
BCTIV-C [IR:Nesh:115:Bean:10]	JX45808	*Phaseolus vulgaris*	Khorasan Razavi (NE)	[5]
BCTIV [IR:Oru:7B:Sug:10]	JX94557	*Beta vulgaris*	West Azarbayejan	[5]
BCTIV-D [IR:Tabr:8RB:Sea	JX94557	*Beta vulgaris*	East Azarbayejan	[5]
TCTV [IR:Zaf:Z5-2:Tur:12]	KC1089	*Brassica rapa var.*	Fars (S)	[22]

Comparing the putative translation products of BCTIV-Kaf genes with those of the BCTIV isolates from various hosts and geographical regions available in GenBank (Table 2) revealed a high degree of conservation for C1 (93.3-100 %), C2 (95.0-100 %) and V1 (88.8-100 %) proteins. However, the two other virion-sence ORFs, V2 (60.9- 98.3 %) and V3 (77.5-98.8 %), were more variable (Table 3). Interestingly, V2 and V3 ORFs are the least similar ORFs (V2 with 60.9 % and V3 with 77.5 % aa similarity) between BCTIV-Kaf and a bean isolate of BCTIV. In addition, there is number of unique substitutions for amino acids in V2 and V3 of BCTIV-Kaf. In the V2 protein, Valine33 substituted with Lysine and in the V3 protein, Glycine58 was substituted with Alanine in other reported BCTIV isolates.

**Figure 3:**
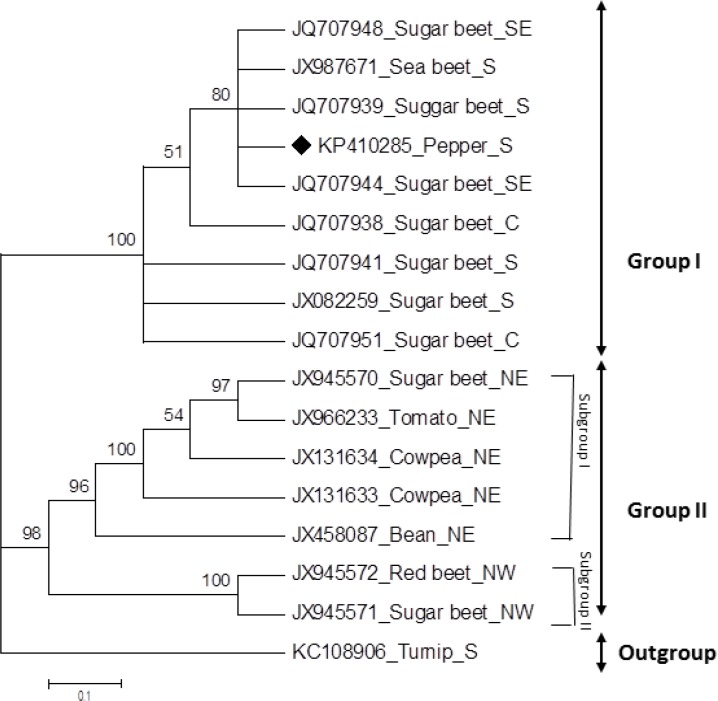
Phylogenetic tree obtained from the alignment of nucleotide sequences of full-length genome of 16 BCTIV isolates from various hosts and geographical regions in Iran. The tree was obtained using the Neighbor-Joining method [22] and MEGA 6 program [23]. The bootstrap analysis is supported with 1000 replications. Branches corresponding to partitions reproduced in less than 50% bootstrap replicates were collapsed. BCTIV-Kaf (KP410285) indicated by a black rectangle. *Turnip curly top virus *(KC108906) was also used as an out-group. The abbreviated letters shows geographical regions in Iran (SE: southeast; S: South; C: center; NE: northeast; NW: northwest).

A dendrogram (Fig. 3) obtained by phylogenetic analysis of the full-length DNA genome sequence of BCTIV-Kaf and other BCTIV isolates from various hosts and geographical regions whose data was available in GenBank (Table 2), revealed two main groups. The first main group (Fig. 3, Group I) is represented by isolates of BCTIV from south, southeast and central regions of Iran while the second main group (Fig. 3, Group II) encompasses BCTIV isolates from the north regions of Iran. This main group was divided into two subgroups. The first subgroup is represented by BCTIV isolates of various hosts from northeast region of Iran and the second subgroup contains only two BCTIV isolates, one sugar beet and one red beet isolate, both from northwest regions of Iran. BCTIV-Kaf isolated from pepper in this study grouped with sugar beet isolates along with a sea beet and sugar beet isolates, all from south, central and southeast regions in Iran. This analysis showed that variation of BCTIV is based more on geographical region rather than host plants.

**Table 3:** Sequence similarity between BCTIV-Kaf and the selected BCTIV from various hosts and geographical regions

Percent identities*			nucleotide			Amino acids		
Host	Geographical	GenBank	Full	V1	V2	V3	C1	C2
	North East	JX945570	90.1	93.2	86.7	86.5	94	98
	North West	JX945571	88.2	88.8	81	88.7	93.3	96
	Central	JQ707938	99	100	98	96.6	100	100
	South	JQ707939	99.2	100	97.5	96.6	100	100
*Beta vulgaris*	South	JQ707941	98.3	100	98.3	98.8	97.3	100
	South East	JQ707944	98.8	99.2	96.6	97.7	99.3	100
	Central	JQ707951	98	99.2	93.3	95.5	98	100
	South	JX082259	98.7	100	98.3	97.7	98.3	100
	South East	JQ707948	99.3	100	96.6	97.7	100	100
*Beta vulgaris subsp.*	South	JX987671	98.6	100	95.8	97.7	97.3	100
*Maritima*	North West	JX945572	88.3	88.8	81.8	89.8	94	95
*Solanum lycopersicum*	North East	JX966233	90.1	93.2	87.6	86.5	94.3	98
*Vigna unguiculata *North East		JX131633	90	92.8	86.7	86.5	93.7	98
	North East	JX131634	90.3	93.2	86.7	86.5	94	98
*Phaseolus vulgaris*	North East	JX458087	88	90.8	60.9	77.5	94.3	99
Mean similarities**			94.32	95.9	89.7	92.02	96.5	98.8

* Percent identity by amino acid sequence of putative ORFs from GenBank

** The mean similarities indicate the mean percent identity shared with reported BCTIV from the GenBank database

To test the infectivity and fulfill the Koch’s postulates of the BCTIV-Kaf genome, a partial dimeric (1.4 mer) clone was constructed in a binary vector to form pBin20- 1.4BCTIV-Kaf. Pepper (bird chili, *C. frutescens*, a local variety), tomato (*S. lycopersicum *cv. Grosse Lisse) and *N. benthamiana *seedlings were each agroinoculated with 5 µl of bacterial suspension (OD600=0.2) containing the pBin20-1.4BCTIV-Kaf construct.

**Figure 4:**
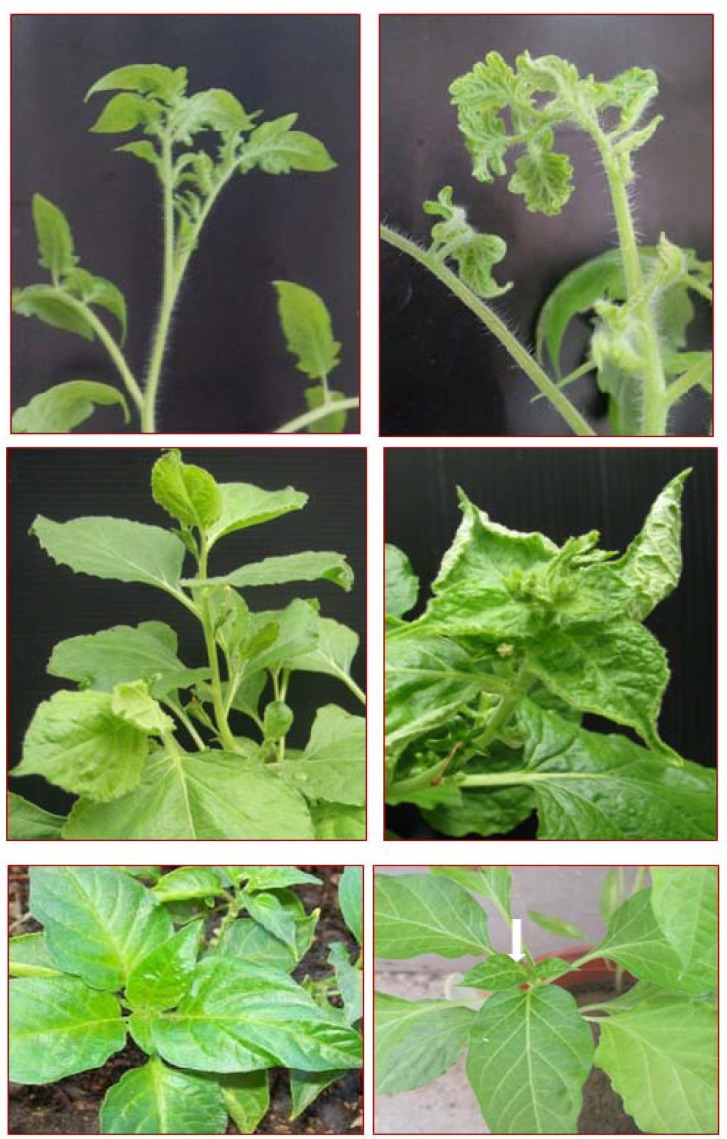
Symptoms induced by BCTIV-Kaf infectious clone in experimentally agroinoculated tomato (A, right) and *Nicotiana benthamiana *(B, right) plants showing reduced size of young leaves, inward rolling of the leaf margins, leaf curling and reduced plant growth 14 dpi compared to a healthy tomato plant (A, left) and a healthy *N. benthamiana *(B, left). A BCTIV-agroinoculated bird chili (C, right) seedling showing mild symptoms including pale green leaves and vein yellowing of young leaves (indicated by arrow) compare to a healthy bird chili seedling.

**Figure 5:**
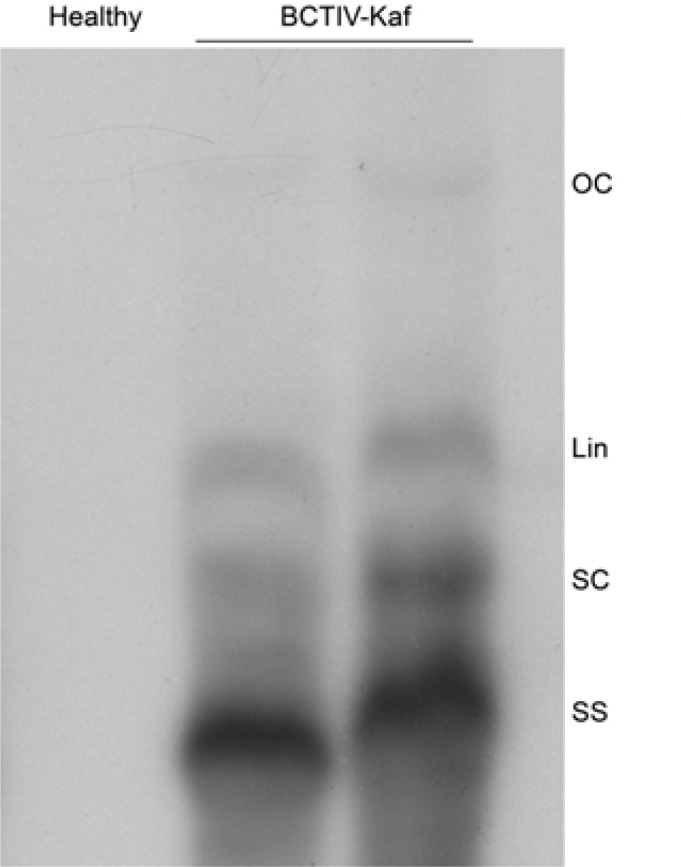
Southern blot analysis of total nucleci acids of a healthy *N. benthamiana *(lane 1) and two separate *N. benthamiana *plants (lanes 2 and 3) agroinoculated with pBin20-1.4BCTIV-Kaf infectious construct hybridized with a 32P-labelled BCTIV probe. The positions of single-stranded (SS) DNA and open circular (OC), linear (lin), supercoiled (SC) dsDNA replicative-forms are indicated. Each lane was loaded with approximately 5 µg of total DNA

Agroinoculated tomato and *N. benthamiana *plants exhibited reduced size of young leaves, inward rolling of the leaf margins, leaf curling and reduced plant growth 14 dpi (Fig. 4A and B). Symptoms were more severe in *N. benthamiana *(Fig. 4B) compared to moderate symptoms in the tomato (Fig. 4A), whereas the bird chili (*Capsicum frutescens*) seedlings, of a local variety used in this study, showed mild symptoms including pale green leaves and vein yellowing of young leaves (Fig. 4C). Systemic infection and replication of the inoculated virus were confirmed by Southern blot assays using total DNA extracted from the newly emerged leaves at three weeks after inoculation. A representative Southern blot is shown in Fig. 5, in which both the predominant viral ssDNA and the dsDNA replicative forms of the virus are visible.

## DISCUSSION

A new variant of BCTIV from pepper plants, showing yellowing and leaf curling symptoms, was isolated and characterized in this study. Agroinoculation of tomato, pepper and *N. benthamiana *plants in greenhouse with the infectious clone of this BCTIV variant produced the typical and severe curly top symptoms (Fig. 4). However, the agroinoculated seedlings of a local variety of pepper displayed mild symptoms (pale green leaves and vein yellowing of young leaves) which were quite different than the observed severe symptoms (yellowing, stunting, shortening of the leaf nodes, leaf curling and upward leaf rolling of leaves) of BCTIV- infected pepper plants from fields in the Kaftarak region. If BCTIV is considered as the only pathogen causing the symptoms in the farm plants, the differences in symptoms may be due to the different pepper varieties used in the agroinoculation experiment at the greenhouse versus the varieties cultivated on pepper farms. It seems that different varieties of pepper used in the field and the greenhouse show different reaction to the virus. Proof of this hypothesis requires further investigation in the field and the greenhouse. Another possibility is that the BCTIV is not the only causal agent of the yellowing and leaf curling disease of pepper plants on farms. Other viruses may be involved in causing disease. It should be noted that pepper farms in Kaftarak region were severely infected by whitefly *Bemisia tabaci*, the unique vector of begomoviruses. The possible role of these viruses in the etiology of the disease on the farms should be taken into consideration.

After using the infectious clone of BCTIRV-Siv (a suger beet isolte of BCTIV) in agroinoculation experiments on different plant species, Soleimani *et al. *(2013) reported that this virus appeared only poorly adapted to sweet pepper as well as tomato and Jimson weed, in which infection rates were low. However, sugar beet, spinach, *N. benthamiana *and *A. thaliana *were highly susceptible to this virus. Different geminiviruses were reported to be associated with curly top diseases around the world. In addition to BCTIV, a severe isolate of BCTV (BCTV-Svr) was detected in pepper farms in the Kaftarak region [17]. A highly divergent strain of *Beet mild curly top virus *(BMCTV), was found to be associated with an outbreak of curly top disease in peppers in Mexico [18]. In the same region (Mexico), synergistic interaction between *Pepper huasteco yellow vein virus *and *Pepper golden mosaic virus *was observed in mixed infection of pepper to these begomoviruses [19]. Other geminiviruses including *Pepper curly top virus *(PCTV) and *Pepper yellow dwarf virus *(PYDV) were also reported in chili peppers in New Mexico [20]

Considering the sequence of the full genomic DNA, and following the ICTV guidelines [6], BCTIV-Kaf is classified as a new variant of BCTIV detected in natural pepper plants, and named BCTIV-[IR:Kaf.2016]. Our phylogenetic analyses (Fig. 3) indicated that BCTIV isolates are grouped based on their geographic distribution rather than their host species. BCTIV-Kaf grouped with BCTIV isolated from sugar beet and sea beet from the same geographical regions, south and central regions of Iran. Meanwhile isolates derived from different hosts such as sugar beet, red beet, tomato, bean and cowpea, all from north region of Iran, clustered in another group distinct from the South and central isolates cluster. A sugar beet and a red beet isolate from North West regions of Iran constituted a subgroup in north group close to the another subgroup including isolates from the northeast region (Fig. 3) confirming that BCTIV isolates show mostly geographical adaptation by region rather than host range adaptation. Consistent with these results, Gharouni Kardani et al [5] reported that BCTIV isolates from northeast and northwest Iran were encompassed in two close subgroups that clearly distinct from BCTIV isolates from the southern regions [5].

Comparing the amino acid sequences of BCTIV-Kaf genes with counterparts of other BCTIV isolates showed that the C1, C2 and V1 proteins had the highest levels of similarity. In contrast, the amino acid sequences of the V2 and V3 proteins were more variable (Table 3). This supports the conserved and key function for C1 and C2 proteins and V1 protein in replication and encapsidation of becurtoviruses, respectively [4].

Since the early report of BCTIV from southern provinces of Iran [11], this important pathogen has spread very rapidly and it is now quite prevalent in Iran. BCTIV has been considered as a dominant and widespread curly top producing agent in important crops in Iran [5, 9, 11]. Epidemics of curly top disease in dicotyledonous crops especially sugar beet, tomato and pepper have occurred annually causing devastating damage to both field and greenhouse crops in recent years. Possible presence of other molecular variants and strains of the virus may further shed more light on its taxonomy, spread and role of this virus in curly top epidemics. The diversity and wide occurrence of BCTIV creates a big challenge for breeders to produce plants with resistant or tolerant traits [21]. Availability of BCTIV infectious clones, including the infectious clone constructed in this study, would be useful tools for screening host plants for their reaction/resistance to this important virus.
